# Visual categorisation of images of familiar objects based on their authenticity: an fMRI study

**DOI:** 10.1007/s00221-024-06989-3

**Published:** 2025-03-10

**Authors:** Grace A. Gabriel, Cristina Simões-Franklin, Georgia O’Callaghan, John Stapleton, Fiona N. Newell

**Affiliations:** School of Psychology and Institute of Neuroscience, Trinity College Dublin, Dublin, Ireland

**Keywords:** Real, Fake, CoS, Texture, Object perception, Categorisation

## Abstract

Under most circumstances, we can rely visual information to quickly and accurately discriminate “real” objects (e.g., fresh fruit) from “fake” objects (e.g., plastic fruit). It is unclear, however, whether this distinction is made early along the ventral visual stream when basic object features such as colour (e.g., primary visual cortex; V1) and texture (e.g., collateral sulcus; COS) are being processed, or whether information regarding object authenticity is extracted in later visual or memory regions (e.g., perirhinal cortex, lateral occipital cortex). To examine this question, participants were placed in an fMRI scanner, and presented with 300 objects photographed in colour or greyscale. Half of the objects were fake, and the other half were real. The participant’s task was to categorise each image as presenting either a real or fake object. Broadly, our analyses revealed significant activation in CoS when participants categorised real objects, particularly when they were presented in colour. We also observed activation in V1 for coloured objects, particularly real ones. These results suggest that our seemingly intuitive ability to rapidly discriminate real from fake objects occurs at the early stages of visual processing, such as when the brain is extracting surface-feature information like texture (CoS) or colour (V1). Future studies could consider the time course of these neural events and probe the importance of cross-modal (e.g., audition and haptic) information underpinning feature extraction for distinguishing real from fake objects.

**Supplementary Information:**

The online version contains supplementary material available at 10.1007/s00221-024-06989-3.

Our brains constantly process large amounts of rich, multisensory information as we explore and interact with our environments. For instance, when deciding whether a piece of bread is fresh or stale, we might look to see whether there have been any changes in its colour, we might try and assess its freshness based on odour, taste a piece to evaluate potentially mustiness, or even use tactile cues to gauge temperature or tenderness. In this way, sensory information is not often used in isolation (Alais and Burr 2004; Ernst and Banks 2002; Ernst and Bülthoff 2004; Meredith and Stein 1986). Nor is it likely that information from the environment is learned or perceived by any one sense in isolation. Thus, prior knowledge can play a role such that perception in one individual sense may be based on predictions from the information provided from other senses (Newell et al. 2001, 2003). Ultimately, while some senses may be better suited for providing information regarding specific attributes of an object (e.g., surface texture via touch, shape via vision; Freides 1974; Welch et al. 1980), our tactile experience from handling objects can offer useful cross-modal predictions for vision thereby facilitating more accurate estimates of an object’s features in the absence of additional corroborating sensory information (e.g., judging fluffiness visually, without touching the object; Whitaker et al. 2008b; Woods and Newell 2004). In other words, the simultaneous encoding of visual and haptic cues, which are learned over time, may allow the brain to infer, or make predictions about, visual information based off of haptic cues, and vice versa (Körding et al. 2007; Sato et al. 2007; Wozny et al. 2010).

Here we examined the role of visual features on the brain’s ability to make categorical judgements about the authenticity of a given item (real versus fake, or natural versus synthetic) using images of everyday objects. In other words, we planned to present participants with images of “real” fruit, flowers, or dessert, as well as images of their fake counterparts (e.g., plastic, crocheted). Importantly, however, we acknowledge that images of a given object carry less information than their real-world tangible counterparts. Specifically, 2D images usually provide only monocular cues to depth, lack affordance (i.e., the potential that a person can perform a genuine action on the item such as grasping it), as well as access to multisensory information such as sound, temperature or odour (Gomez et al. 2017; Snow et al. 2014; see Snow and Culham 2021 for a review). Thus while these factors make images an unusual class of stimuli for studying object perception, the ultimate goal of this study was to determine the neural underpinnings of visual object categorisation for ‘real’ versus ‘fake’ (i.e., natural versus synthetic) items. In this case, using images rather than tangible items allowed us to control for potential access to additional (i.e.., multisensory) information, that could influence relevant neural processing or participants’ decision-making when evaluating the objects. For instance, participants may be better able to distinguish real from fake fruit, flowers, or dessert via scent, but images of these objects would not carry such additional, corroborating information. By relying on only visual stimuli via images, we could better examine the neural responses to inherent visual characteristics that distinguish real from fake objects, without the influence of confounding cues.

Intuitively, our ability to distinguish real objects from fake objects visually, might stem from our capacity to discriminate between surface features, including their material. Indeed, humans can discriminate a wide range of object materials, using only visual cues (Fleming 2014). As such, neural regions which code for features that are necessary for identifying materials – like texture and colour – may be recruited during real and fake object categorisation. If this is the case, then one suggestion might be that early sensory regions processing these low-level visual features might provide sufficient information to allow real and fake objects to be distinguished from one another. For instance, the ability to determine whether an apple is real or fake may depend on early visual processing of the apple’s texture. If this is the case, then we might expect to find that the ability to distinguish real from fake objects would recruit regions previously identified to code for texture, along the collateral sulcus (CoS; Cant et al. 2009; Smith and Goodale 2015). Neuroimaging studies have shown that visual textures are represented independently in many brain areas, and are generally spread across the visual pathway, with an emphasis on the fusiform gyrus, lingual gyrus, and in particular primary visual cortex as well as collateral sulcus (CoS) (Cant and Goodale 2011; Hiramatsu et al. 2011; Peuskens et al. 2004; Podrebarac et al. 2014; Whitaker et al. 2008b).

The perception of object authenticity may also rely on other lower-level features such as colour. Colour is a feature known to allow for faster identification or categorisation of scenes, compared to when those same scenes are presented in black-and-white (Gegenfurtner and Rieger 2000), particularly when the colour feature is relevant to the meaning of the scene (Oliva and Schyns 2000). Colour has also been shown to be an important feature for object and material identification (Witzel and Gegenfurtner 2018), including identifying real objects such as fruit from a distance (Bompas et al. 2013). Interestingly, some research suggests that colour can benefit the identification of specifically, natural, but not non-natural objects (Humphrey et al. 1994).

Evidence from patient studies suggests that the processing of visual features such as colour might involve specific regions of the occipital and temporal lobes including the fusiform gyrus and lingual gyrus (James et al. 2003; Meadows 1974; Steeves et al. 2004; Zeki 1990; Zihl and Heywood 2016). These results were later corroborated by neuroimaging studies in healthy participants (Zeki et al. 1991). Other neuroimaging studies in healthy populations also show activation in occipital (V1) and more dorsal brain regions like the cuneus (Beauchamp et al. 1999; Cant and Goodale 2007) which is consistent with previous evidence suggesting that colour is processed early in the visual pathway.

However, while surface features may contribute to some information about an object’s material, this information may not be sufficient for the brain to visually categorise an item as real or fake. Instead, we do not rule out the possibility that more anterior regions may support the ability to determine the authenticity or naturalness of a given object. For instance, many regions along the ventral visual stream (VVS) have been implicated in object recognition (Grill-Spector et al. 2001; Grill-Spector and Malach 2004; Mahon and Caramazza 2009; Op De Beeck et al., 2008), most notably, the lateral occipital cortex (LOC). The LOC has been found to be sensitive to the basic features and overall shape defining an object’s characteristics (Grill-Spector et al. 1999, 2000; Haxby et al. 1991, 2001) It contains subregions including LO (a region which overlaps with LO-2) as well as posterior fusiform regions (pFus; which overlaps with the posterior fusiform gyrus; Grill-Spector and Malach 2004; Kravitz et al. 2013), and appears to be organised topologically (Grill-Spector and Weiner 2014). Still, while LOC might work to distil information received earlier in the VVS via feedback loops, there is little evidence to suggest it codes for object categories per se – only object shape (Bracci & Op De Beeck, 2022; Kourtzi and Kanwisher 2001). Instead, other regions in occipitotemporal cortex (OTC) which overlap with LOC appear to code more readily for object categories. In fact, much of the literature on cortical specialisation point to regions in the OTC (Rosenke et al. 2021), including regions with selective sensitivity for faces (fusiform face area; Gauthier et al. 2000; Kanwisher and Yovel 2006; Palejwala et al. 2020; Sayres and Grill-Spector 2008), scenes (parahippocampal place are; Epstein 2008; Epstein and Kanwisher 1998; Palejwala et al. 2020; Sayres and Grill-Spector 2008), and words (visual word form area; Dehaene and Cohen 2011; McCandliss et al. 2003). However, most object *categories* do not seem to have their own distinct neural regions and seem instead to be processed more broadly in OTC (Bracci & Op De Beeck, 2022). Therefore, it is possible that authenticity (i.e., real versus fake) categorisation is also processed in regions beyond the LOC, including the OTC.

One final consideration is the possibility that the multisensory features used to determine an object’s authenticity are learned over time and thus feedback from object representations in memory regions of the brain may support the categorisation of object images as real or fake (Cromer et al. 2010; Gilbert and Li 2013). For instance, studies of patients with medial temporal lobe (MTL) damage – particularly when it extended into perirhinal cortex (PRC) – suggests that PRC is heavily implicated in object discrimination as well as recognition (Barense et al. 2010; Cowell et al. 2006; Murray et al. 2007), and may play an important role in object categorisation. Located at the boundary between the medial temporal lobe and the VVS, the PRC is thought to integrate sensory information into coherent representations of objects (Barense et al. 2007; Lee and Rudebeck 2010), and its sensitivity to fine-grained differences between objects may be useful in determining an object’s authenticity. Specifically, the PRC appears to play a significant role in distinguishing among perceptually and semantically similar or confusable, items (Bruffaerts et al. 2013; Holdstock et al. 2009; Newell et al. 2023). Thus, the PRC may be important for identifying the subtle differences separating fresh from plastic flowers, for example. Even more critically, the PRC appears to be involved during congruent visual-tactile cross-modal object matching (Holdstock et al. 2009), and while particularly responsive to visual information, is modulated by cross-modal learning (Li et al. 2024). It is therefore possible that activation in PRC might reflect learned cross-modal correspondences between the perceived visual texture, and assumed tactile texture, thereby supporting object authenticity categorisation (e.g., viewing the previously learned haptic texture of an object, using only vision). Therefore, we might predict that real versus fake category discrimination may be mediated by more anterior regions like the PRC, that are not only involved in binding sensory information (Barense et al. 2010; Murray et al. 2007) but are also critical for processing finer category differences or details (Kivisaari et al. 2012; Tyler et al. 2004).

Still, PRC activation may only be observed when participants engage in tasks requiring fine-grained discrimination of objects since its involvement would be crucial for these detailed tasks, whereas more general discrimination tasks may not sufficiently engage the PRC to the same extent (Bussey and Saksida 2005; Cowell et al. 2006; Tyler et al. 2004). Therefore, to effectively capture PRC activation, we might need to use object stimuli that are highly similar and a task that involves fine grained discrimination, and that we may not necessarily observe more activation when processing specifically real objects, or specifically fake objects, during the task *per se.* Thus, while we will consider anterior regions via global brain analyses, we expect that more posterior regions near LOC (e.g., near OTC; which may code for broader categories of objects; Bracci & Op De Beeck, 2022) as well as earlier visual regions like CoS and V1 to be more likely candidates as neural regions supporting real versus fake object categorisation.

## Current study

In our study, we were specifically interested in elucidating what region(s) of the brain were sensitive to real and fake categories of objects and whether this categorical distinction arose early on, in sensory regions of the brain such as areas sensitive to visual texture or colour (e.g., CoS, V1, LOC) or whether such distinctions would arise in later memory regions (e.g., MTL or PRC). To ensure the ecological validity of our stimulus set, we did not control for the nature of the texture information available across the images of the objects. Nevertheless, we hypothesised that images of real objects may be comprised of more complex textures, when compared to synthetic objects, since there is less uniformity across textures of real objects. As such we expected that brain regions sensitive to texture, such as the CoS, would be particularly responsive to the authenticity of the visually presented objects (Cant and Goodale 2011; Podrebarac et al. 2014; Whitaker et al. 2008b). We also speculated that regions of the brain that process the chromaticity of objects, including early visual areas and regions within the fusiform gyrus (Beauchamp et al. 1999; Cant and Goodale 2007; Steeves et al. 2004; Zihl and Heywood 2016), would respond according to the level of colour information available in the image (colour versus black and white).

To that end, we presented a group of participants with photographic images of familiar objects (adapted from Sharan et al. 2014), half of which were images of natural or real objects (e.g., fresh fruit), and the other half were images of synthetic or fake versions of these objects (e.g., crocheted fruit). For the purpose of our experiment, the object images from each category were presented as colour or greyscale to allow us to further disambiguate the role of colour cues on categorisation. The participants’ task was to quickly judge the authenticity of each visually presented image of these objects, by categorising each image as ‘real’ or ‘fake’. The entire study was conducted within a 3T MRI scanner and participants did not preview the images prior to the scanning session.

While there have been many studies which have examined the role of visual features such as textures for object perception, these studies typically use nonsense or computer-generated (novel) objects and textures (Cant et al. 2009; Cant and Goodale 2007; Cavina-Pratesi et al. 2010). However, there is evidence to suggest that familiar objects encountered in the real-world are processed differently (e.g., remembered better, Snow et al. 2014) or more quickly identified or differently processed in the brain, (Adelson 2001; Bompas et al. 2013; Fleming 2014; Humphrey et al. 1994; O’Callaghan et al. 2018; Snow and Culham 2021) than those which are less realistic (e.g., line drawings, black-and-white images; Balas and Conlin 2015; James et al. 2003; Sharan et al. 2014; Snow et al. 2014). Therefore, what is less clear is how visual cues to the surface properties of familiar, real-world objects such as fruit, food, or flowers, are processed in the brain. More specifically, we were interested in understanding whether the neural substrates underpinning the processing of visual features such as colour and texture are already selective to the perceptual categories of ‘real’ or ‘fake’ or whether such categorisation occurs elsewhere such as in non-sensory regions of the brain.

## Methods

### Participants

A total of 16 participants completed the full protocol of this study and were included in the analyses ($\:{mean}_{Age}$= 21.7 years, *SD* = 1.6, range = 20–24 years). Participants reported no history of neurological, psychiatric, or psychological illness, and had normal or corrected-to-normal vision. This study took place at the Institute of Neuroscience, Trinity College Dublin, and participants were compensated €15 for their time. The duration of the study lasted no more than 1.5 h in total.

### Materials

The stimuli consisted of 300 photographed images of real and fake objects. These photographs were provided by Sharan and colleagues and used in their previous work (2014). All images were cropped to a resolution of 1024 × 768 pixels and were normalized to equate their mean luminance. The photographic images were also pre-screened to ensure that the photographed items were unambiguously rated as “real” or “fake” by a separate set of participants (via 5-point rating scale).

The objects in the images (*n* = 300) belonged to one of three categories: dessert, fruits, and flowers (*n* = 100 objects, in each category). Within each object category, half of the images were of real objects, and the other half, fake. Images were, furthermore, presented in either colour or greyscale. All participants completed the same within-subjects paradigm.

### Procedure

#### Functional task

We used a 3T Achieva Philips fMRI scanner to acquire neuroimaging data. The authenticity categorisation task was presented with Neurobehavioral Systems (NBS) Presentation software (version 0.70, www.neurobs.com) on the central computer of the MR system and participants responded to the task using a 1 × 2 Fibre-Optic response pad. Participants indicated whether they perceived the images to be real or fake using the index and middle fingers on the right hand, with the button assigned to ‘real’ (either middle or index) counterbalanced across participants.

**Fig. 1:**
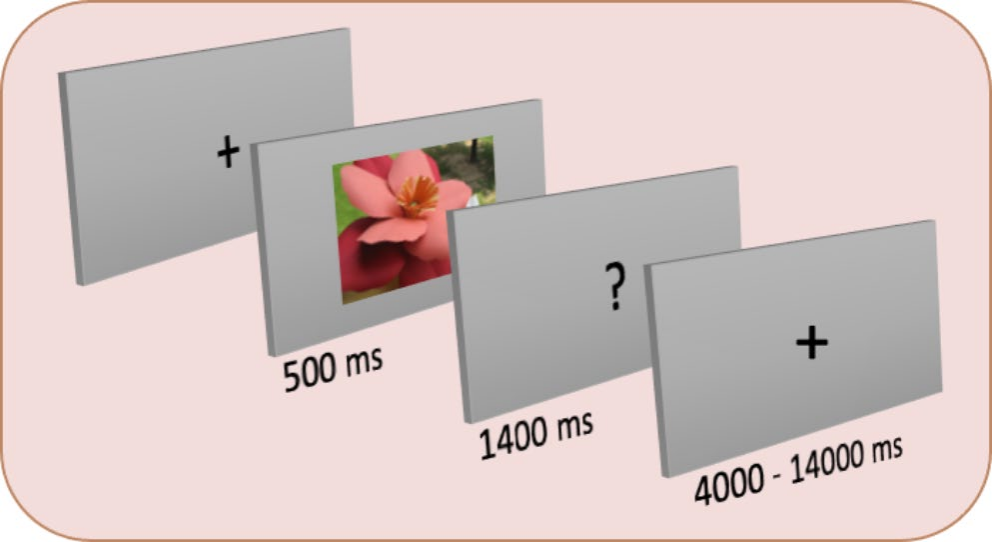
Illustration of a typical trial structure in the object authenticity categorisation task

Participants completed six runs of the authenticity categorisation task, each lasting 320 s (160 TRs). Each run contained 50 trials and, in each trial, participants were presented with a fixation cross, then were presented with an image of one of the randomly selected images for 500 ms. Participants were then given 1400 ms to categorise the image shown in each trial based on their perception (i.e., whether they judged the item to be ‘real’ or ‘fake’). A question mark presented on the screen cued for a response. Inter-trial-intervals (ITIs) jittered between four and 14 s (fixation cross). An overview of the task is illustrated in Fig. 1.

**Fig. 2:**
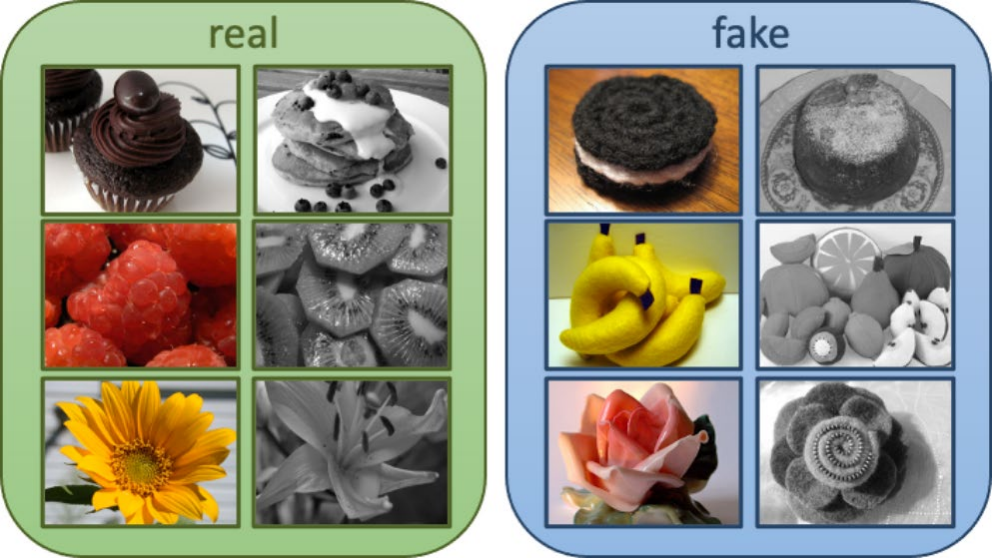
Examples of the six types of images of objects included in the authenticity categorisation task. Either colour or black and white images of'real' (left panel) and'fake' (right panel) versions of desserts, fruit and flowers were presented

In total, across the six runs, 300 images were presented: 25 each of real fruit, flowers and desserts in colour, real fruit, flowers and desserts in black and white (B&W), fake fruit, flowers and desserts in colour and fake fruit, flowers and desserts in B&W. The six possible image types are illustrated in Fig. 2. Four versions of the task were designed to counterbalance the order the images were presented in either colour or B&W and, as previously mentioned, which finger was used to indicate a'real' or'fake' response, across participants.

The images chosen for this study were independently validated in a pilot rating-study with 16 different participants (see Supplemental Materials). In this pilot study, participants rated each image on a 7-point scale, ranging from “very synthetic” to “very natural”. Statistical analyses did not reveal any significant effects or interactions involving image type (fruit vs. flowers vs. desserts). Therefore, it was deemed that, regardless of colour or image type (i.e., fruit, flowers, or desserts) participants were able to correctly identify images that contained depictions of'real' or'fake' objects. Thus, images of fruit, flowers and desserts were combined for later analysis of the functional neuroimaging data to increase statistical power.

### Data acquisition

#### Functional magnetic resonance imaging (fMRI)

The current study utilised a Philips Achieva 3.0 Tesla MR system in conjunction with an 8-channel head coil. A mounted mirror reflected a display that was projected onto a panel behind the participant’s head outside the magnet. After an initial reference scan to allow for the resolution of sensitivity variations, 180 high-resolution T1-weighted anatomic MPRAGE transverse images (FOV 230 mm, thickness 1.5 mm, voxel size 1.5 × 1.5 × 1.5 mm, total duration 343s) were acquired for each participant, which allowed for subsequent activation localization and spatial normalization. Functional images consisted of 40 non-contiguous (0.3 mm gap), 3 mm transverse slices covering the entire brain and collected in ascending order. Images were acquired using a T2* weighted echo-planar imaging sequences (TR = 2000ms, TE = 25ms, FOV 240 mm, 80 × 80 matrix size in Fourier space). All imaging utilised a parallel sensitivity encoding (SENSE) approach with a reduction factor of 2.5 (Pruessmann et al. 1999).

#### fMRI preprocessing and statistical analysis

Analysis of fMRI data was conducted using MATLAB 2016a (“MATLAB: MATLAB and Statistics Toolbox Release,” 2016a) and using Statistical Parametric Mapping, version 12 (SPM12, 2014). The imaging data underwent the following preprocessing steps using the default settings: realigning all scans across the six runs to the first scan of the first run, coregistration to structural images, segmentation, spatial normalisation (during which the data were resampled to 2mm^3^ voxel dimensions) and smoothing with a Gaussian kernel of full-width half maximum (FWHM) 8mm^3^. Volumes with scan-to-scan motion in excess of 1 mm were identified by the ArtRepair toolbox (by Mazaika et al. 2009) and were flagged to be deweighted in the design matrix phase.

Individual level analyses on the task were performed using the general linear model (GLM), implemented in SPM12. All six runs were included in the design matrices but entered as separate sessions. These matrices included a column for the onsets of each of the four types of stimuli^1^ (real colour, real B&W, fake colour, and fake B&W), a regressor to deweight excessive motion, where necessary, and the six motion parameter files produced from realignment. With the exception of excessive motion (for which the durations was entered as two seconds = 1 TR), all events were modelled as zero duration events. Designs were estimated using an autoregressive (AR(1) level autocorrelation) model and the HRF was determined using the canonical approach with no derivations. A high pass filter (HPF) of 128 Hz, the default in SPM12, was also applied in all instances.

First level analysis consisted of contrasting each of the four conditions (real colour, real B&W, fake colour and fake B&W) against baseline. Following this, the flexible factorial approach in SPM12 was used to apply a 2 × 2 within-subjects ANOVA for authenticity (fake vs. real) by colour (colour vs. B&W) for these first level contrasts at the group level, with correct and incorrect events modelled separately. Three factors were entered in the following order: subjects, authenticity, and colour. Independence and equal variance were assumed for ‘subjects’, whereas authenticity and colour were assumed to have unequal variance and not to be independent. Main effects were produced for the three factors, in addition to an interaction between factors two and three. Results were reviewed with *t*-contrasts with an uncorrected threshold of 0.001 at the voxel level but had to exceed a Family-Wise Error (FWE) corrected cluster size of 230 (1840 µl) continuous voxels, determined by AFNI’s 3dClustSim program (Analysis of Functional NeuroImages) with the following settings: probability of a cluster < 0.01; voxel threshold *p* <.001; 10,000 iterations; FWHM of 9.5 mm^3^; one-tailed; faces touching, AFC values = 0.388767, 5.23473, 11.3208. Results are summarised in Table 1 and include MNI coordinates of peak activations. Therefore, while these coordinates are reported for reference, cluster-wise corrections were applied and the interpretation of the data is based on cluster-level significance rather than individual peak voxels.

**Table 1:** Summary of the results of the whole brain analysis of the 2 × 2 within-subjects ANOVA of authenticity (real vs. fake) by colour (colour vs. B&W). Peak voxel coordinates are in MNI space

	Cluster extent (k)	Cluster volume (µl)	Cluster *p*(FWE-corrected)	Peak voxel T	Peak voxel coordinates (x, y, z)	Peak locations
*Real > Fake*						
Cluster 1	398	3,184	0.000	5.52	2, -30, 74	Right M1
				3.97	2, -28, 64	
				3.59	-4, -26, 84	Left M1
Cluster 2	333	2,664	0.000	5.38	-34, -28, 56	Left M1
				4.06	-28, -34, 56	Left S1
				3.87	-14, -40, 58	Left sensory association cortex
Cluster 3	781	6,248	0.000	5.21	0, 4, 46	Right pre-motor and supplementary motor cortex
				4.34	-14, -2, 50	Left pre-motor and supplementary motor cortex
				4.07	2, 20, 40	Right FEF
Cluster 4	1163	9,304	0.000	4.98	8, -82, 0	Right V1
				4.77	20, -90, 24	Right visual association cortex
				4.35	26, -80, 24	
Cluster 5	716	5,728	0.000	4.34	-14, -98, 0	Left secondary visual area (i.e., parastriate incl. LO gyrus)
				4.22	-38, -84, 16	Left visual association area (incl. LOC)
				4.18	-10, -86, -2	Left secondary visual area
*Fake > Real*						
Nothing significant						
*Colour > B&W*						
Cluster 1	447	3,576	0.000	7.23	22, -48, -4	CoS
				4.93	26, -76, -8	
Cluster 2	349	2,792	0.000	5.77	-24, -52, -6	CoS
				3.64	-26, -34, -6	
*B&W > Colour*						
Nothing significant						
*Interaction*						
Nothing significant						

#### Mean activation extraction and analysis

For two regions of interest (ROIs), the bilateral collateral sulcus (CoS) and primary visual cortex (V1), mean activations were extracted through the eigenvariate function in the results viewer of SPM12 and exported to SPSS for follow up analysis. For these regions, a 15 mm sphere was placed around the peak-voxel in each hemisphere during display of the main effects of colour and authenticity, respectively. This meant that signal was extracted for the CoS from MNI coordinates − 24, -52 -6 and 22, -48, -4, and from − 10, -86, -2 and 8, -82, 0 for the V1. Results were summarised to the MATLAB workspace and then copied into SPSS. Consistent with the previous analysis, these mean extracted beta values were subjected to 2 × 2 within-subjects ANOVAs for authenticity (real vs. fake) and colour (colour vs. B&W) and post hoc analyses of interactions with Bonferroni corrections were applied where appropriate.

## Results

### Task performance

Participants’ accuracy at categorising stimuli as either'real' or'fake' on the task was assessed with a 2 × 2 within-subjects ANOVA for authenticity and colour (colour vs. B&W). While main effects of authenticity (*F*(1,15) = 14.52, *p* <.05, η^2^ = 0.509) and colour (*F*(1,15) = 31.80, *p* <.001, η^2^ = 0.694) were found, these should be interpreted in light of a significant interaction between the two factors (*F*(1,15) = 10.34, *p* <.05, η^2^ = 0.425). Post hoc analysis revealed that participants were more accurate at categorising images of objects as'real' when they were in colour compared to images in B&W (*F*(1,15) = 52.64, *p* <.001, η^2^ = 0.79), but colour did not impact on the accuracy of categorising images of'fake' objects (*F* < 1). The relevant means can be found in Table 2.

**Table 2:** Participants’ mean accuracy (and SDs) at categorising the four types of stimuli as either 'real' or 'fake'

		Accuracy
Real	Colour	87.50 (8.75)
	B&W	75.13 (11.64)
	Overall	81.31 (9.75)
Fake	Colour	68.39 (9.1)
	B&W	68.59 (8.79)
	Overall	68.49 (7.361)
Colour		77.95 (5.44)
B&W		71.86 (6.55)
Overall task accuracy		74.25 (6.45)

### fMRI results

#### Whole-brain results

**Fig. 3:**
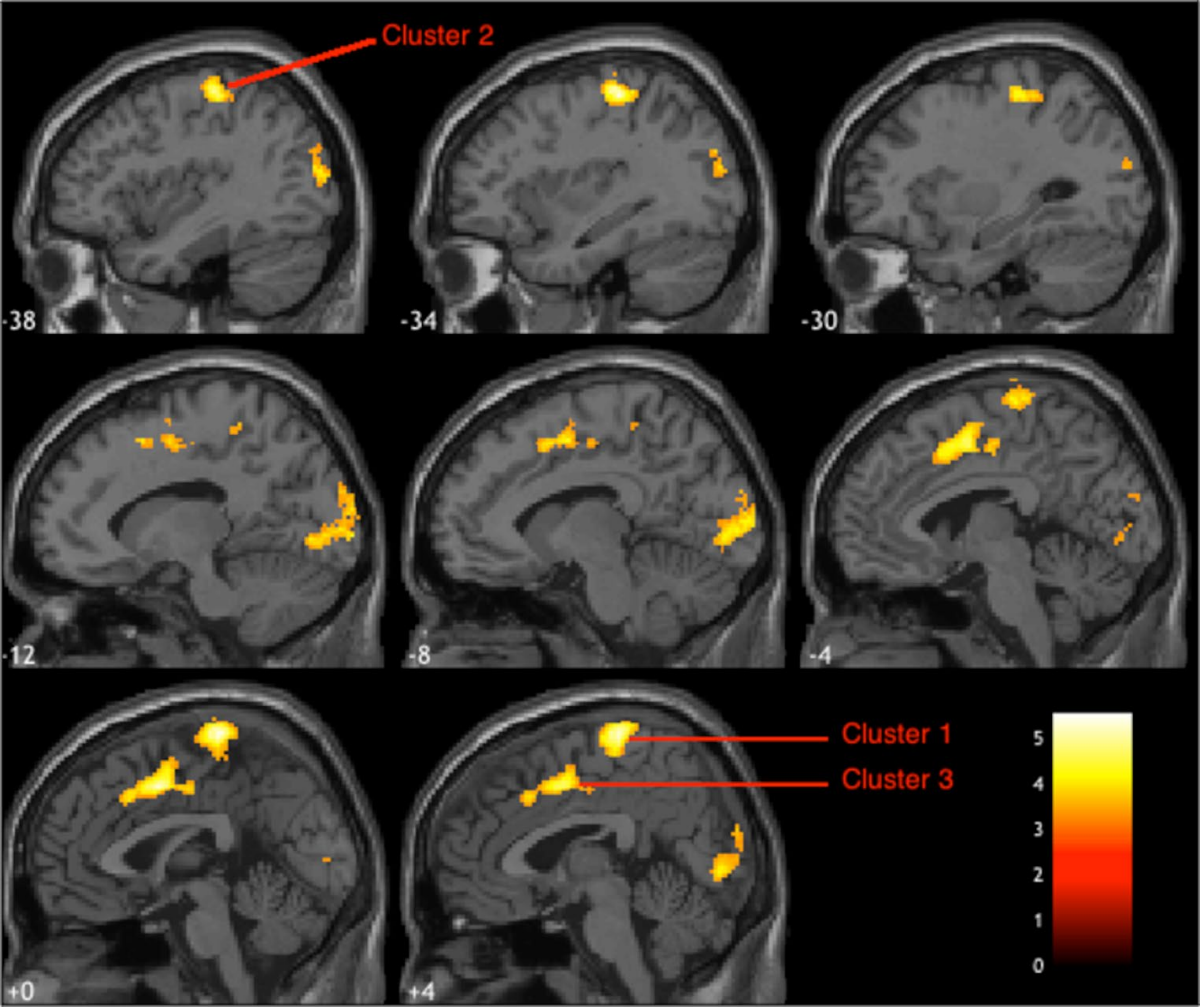
Images of real objects, compared to fake, elicited greater activation in regions of the paracentral lobule (cluster 1), the left pre/post central gyrus (cluster 2) and MCC (cluster 3), illustrated here in sagittal slices. The colour lookup table represents *t*-values

**Fig. 4:**
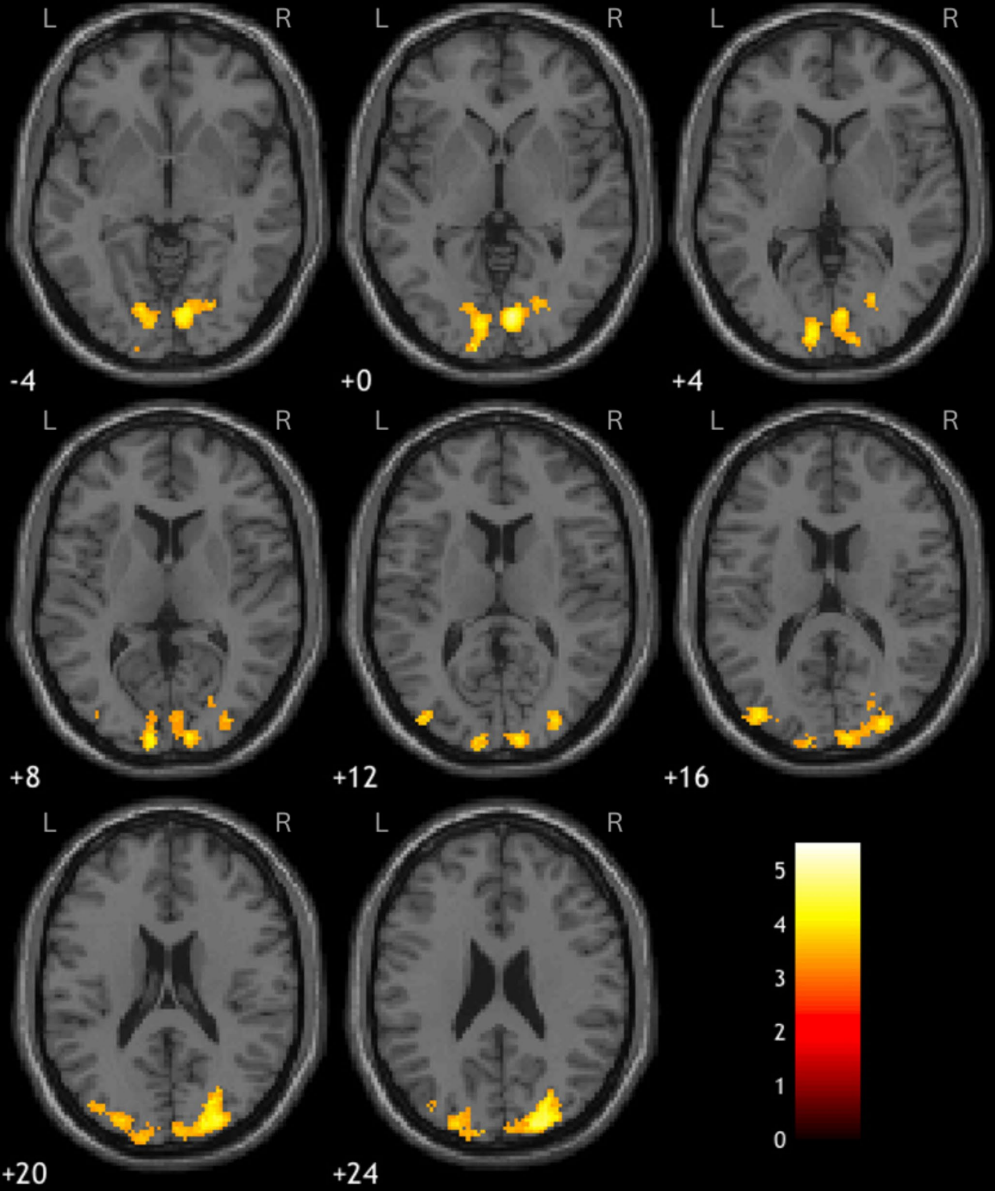
Axial views of the clusters of greater activation observed in early visual areas in response to images of real objects, compared to fake; clusters 4 and 5 in the right and left hemispheres, respectively. The colour lookup table represents *t*-values

**Fig. 5:**
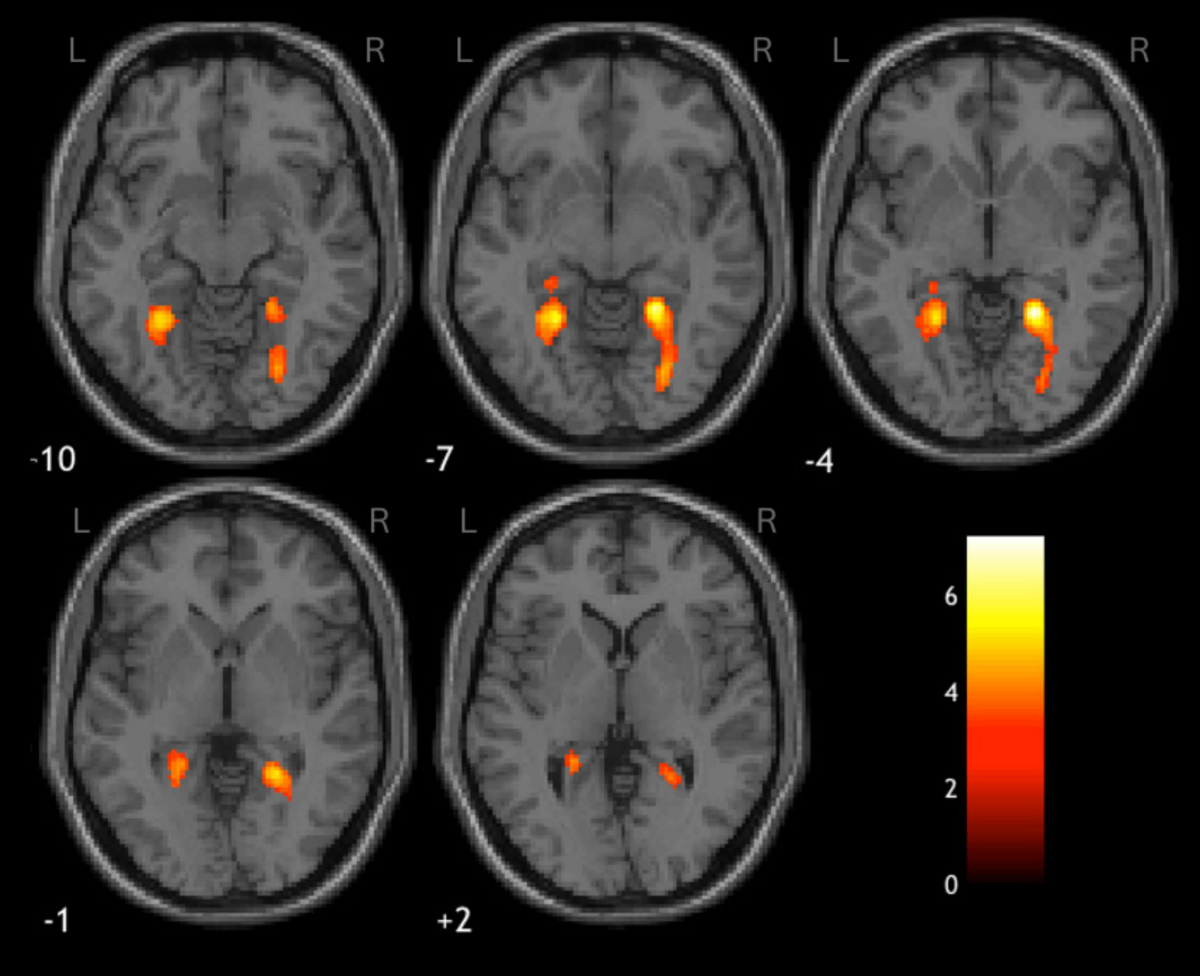
Bilateral CoS recruited in response to coloured over B&W images. The colour lookup table represents *t*-values

Global brain analysis showed that when participants viewed images of real objects, compared to fake, greater activity was observed in early visual areas, in addition to the paracentral lobule, the middle cingulate cortex (MCC) and an aspect of the left pre- and post-central gyri. Furthermore, the bilateral CoS was recruited more when viewing coloured, rather than B&W images. The results of this whole brain analysis are summarised in Table 1 and illustrated in Figs. 3, 4 and 5. Note that some of the clusters listed in Table 1 contain spatially disparate hotspots. These represent larger clusters which span several regions, resulting multiple peaks in activation within a single cluster.

#### ROI analysis

**Fig. 6:**
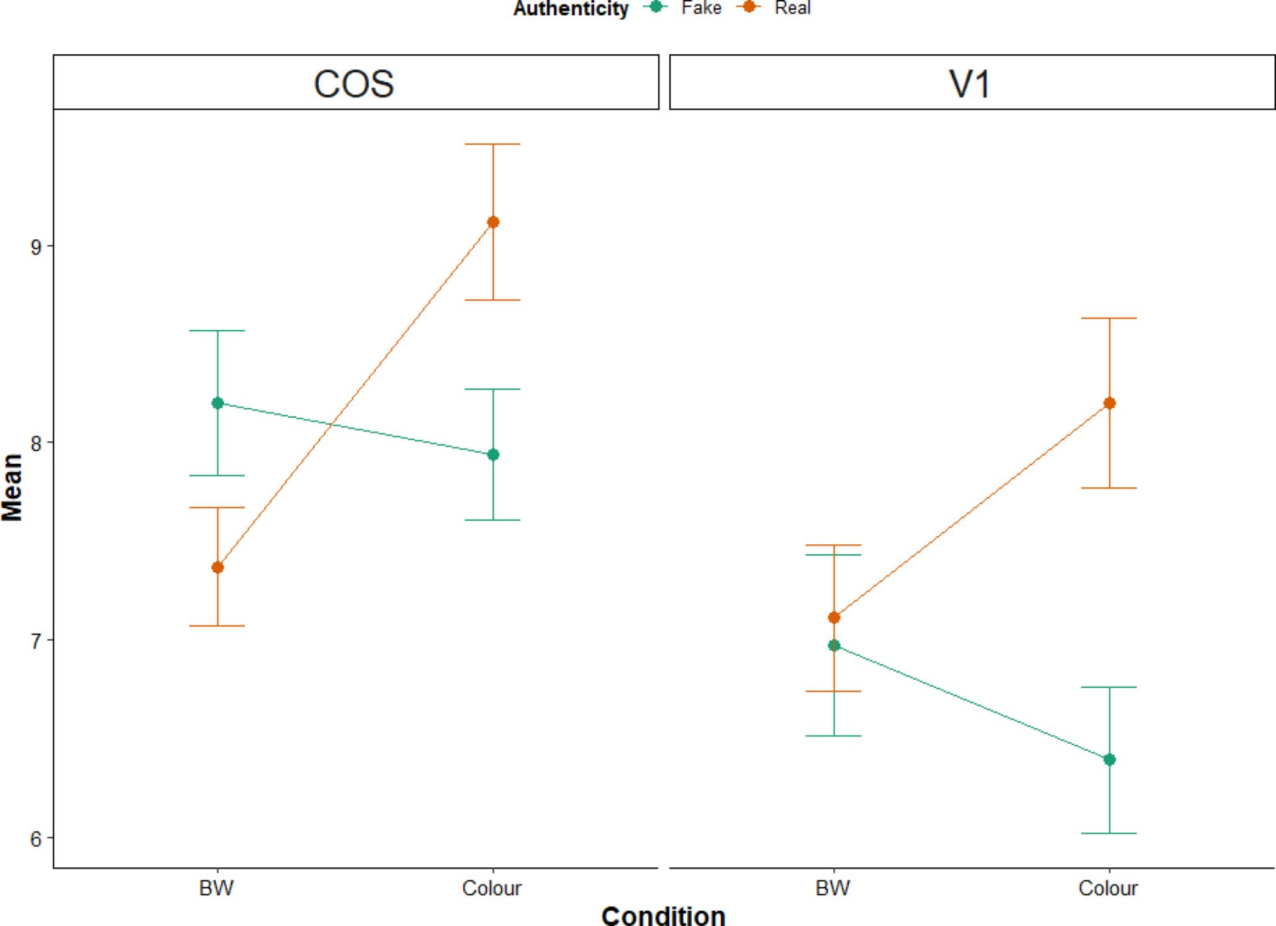
Extracted mean and standard errors from the bilateral CoS and V1 ROIs. See text for details of the interaction

2 × 2 within-subjects ANOVAs for authenticity (real vs. fake) and colour (colour vs. B&W) were conducted on the mean extracted beta values from the bilateral CoS and V1 and post hoc analyses utilised a Bonferroni corrected significance threshold of 0.025 (α = 0.05/2 per hemisphere). Extracted mean values for each condition and region are presented in Fig. 6.

Within the CoS, the data show a significant interaction between colour and authenticity (left: *F*(1,15) = 39.39, *p* <.001, η^2^ = 0.724; right: *F*(1,15) = 41.1, *p* <.001, η^2^ = 0.733), which revealed that the CoS had a greater response to coloured images of real objects compared to B&W (left: *F*(1,15) = 52.53, *p* <.001, η^2^ = 0.778; right: *F*(1,15) = 54.41, *p* <.001, η^2^ = 0.784), whereas there was no effect of colour when viewing fake objects (left: *F*(1,15) = 4.75, *p* >.025; right: *F*(1,15) = 1.12, *p* >.025). See Fig. 5 for details of these activations.

The opposite pattern was observed in the V1: there was a significant interaction between colour and authenticity (left: *F*(1,15) = 13.81, *p* <.01, η^2^ = 0.479; right: *F*(1,15) = 14.96, *p* <.01, η^2^ = 0.499), but in this instance there was a greater response to coloured images of real objects, compared to fake (left: *F*(1,15) = 28.14, *p* <.001, η^2^ = 0.652; right: *F*(1,15) = 55.72, *p* <.001, η^2^ = 0.788), but no effect of authenticity for B&W images (left: *F*(1,15) = 0.076, *p* >.025; right: *F*(1,15) = 0.22, *p* >.025). See Fig. 4 for an example of this pattern of activation.

## General discussion

In this study we aimed to uncover the neural correlates underpinning our ability to visually discriminate real objects from fake objects (e.g., fresh fruit from plastic fruit). Specifically, using a neuroimaging study, participants were tasked with categorising whether a series of colour and greyscale images of objects were real or fake whilst in an MRI scanner. A whole brain analysis showed that early visual regions display an authenticity distinction, with some activation in lateral occipital cortex (LOC) as well as bilateral activity in primary visual cortex (V1), when participants judged ‘real’ (compared to ‘fake’) objects. We also found additional activity in more dorsal regions such as the mid-cingulate cortex (MCC), intraparietal sulcus (IPS), the paracentral lobule, precentral gyrus, and postcentral gyrus. With regards to judging coloured versus greyscale items, participants showed bilateral recruitment of the CoS.

We also conducted ROI analyses on the CoS and V1 consistent with our a priori hypotheses. These revealed that the CoS was particularly responsive to judging specifically coloured objects when real, but not fake. This interaction lends support to previous findings that the CoS is involved in determining authenticity in visually presented objects, and that coloured objects may facilitate the perception of texture (Cant and Goodale 2007; Whitaker et al. 2008a; Witzel and Gegenfurtner 2018). For activations in V1, we observed the opposite pattern: an interaction between colour and authenticity, with greater activity in response to ‘real’ coloured objects, relative to greyscale objects, suggesting the involvement of early visual regions of the brain in colour processing consistent with previous research on the perception of coloured objects (Cant and Goodale 2007; Steeves et al. 2004; Zihl and Heywood 2016).

In the global brain analyses, cluster-wise analyses show activity in sensorimotor areas (Table 1) for real objects relative to fake objects. Indeed, as alluded to earlier, previous reports suggest that visual examination of real, tangible objects is associated with more neural activation in motor regions, relative to images of those same objects (Snow and Culham 2021). These differences in neural activation likely reflect affordances for interaction with the objects (e.g., reaching and grasping) (Gomez et al. 2017). Therefore, it is possible that heightened activation in sensorimotor regions for real (relative to fake) objects in this current study reflect the nuanced differences associated with manipulating a real versus fake objects (i.e., the associated motor plan). For instance, more care may be required when handling real (versus fake) versions of fruit, flowers, or dessert to avoid grasping the wrong area on the object (e.g., petals versus stem), or when calibrating grip force (e.g., apple versus banana).

Within more ventral regions of the brain, we also found evidence of heightened activity in the lateral occipital cortex (LOC) for ‘real’ relative to ‘fake’ items. As previously alluded to, regions in the broader OTC show specialisation for object identification (Bracci & Op De Beeck, 2022), but to our knowledge, have not yet been implicated in the ability to categorise real and fake objects. Critically, the LOC is not usually recruited when processing low-level or basic visual features, including that of object texture (Cant and Goodale 2011; Emberson et al. 2017; Grill-Spector et al. 1999, 2001; Steeves et al. 2004), and as such its role would likely be in extrapolating a combination of relevant features along the ventral visual stream (VVS) when identifying an object. While it is possible that the activity we observed in LOC for “real” objects reflected object identification and familiarity, given its proximity and overlap to other regions in OTC (Bracci & Op De Beeck, 2022), the activation we observed might possibly also reflect activation of more feed-forward connections for categorising'real' from'fake' objects, potentially projected further up along the visual processing stream (Bracci & Op De Beeck, 2022).

With regards to more anterior memory regions, such as the PRC or other regions in the medial temporal lobe, we did not find that such higher order regions responded to the authenticity distinction of the objects. While the PRC has been found to be critical for visual discrimination between highly similar objects (Bussey and Saksida 2005; Tyler et al. 2004), it is possible that such activation might have been apparent had controlled the level of inter-object similarity in the task, in which case we may have found more PRC activation during the authenticity categorisation task, as the PRC is involved in the processing of complex objects (Barense et al. 2007; Diana et al. 2008). In other words, the PRC may be necessary for distinguishing real from fake objects due to the fine-grained perceptual and semantic elements differentiating both categories but might not necessarily mean that we would find more activity for the real relative to fake category processing, or the reverse, especially with already familiar object categories. Instead, earlier regions, which code for broader or more superordinate organisations of object categories may be more relevant to our study’s authenticity categorisation task. Indeed a recent review suggests that activation in the medial temporal lobes is usually associated with inter-category discrimination, and activation differences are not contingent on one specific category over another (Robin et al. 2019). 

Our results suggest that texture may be particularly important for distinguishing an object’s authenticity. As mentioned earlier, we are able to visually discriminate among different textures with high accuracy (Cavdan et al. 2021; Fleming 2014; Wijntjes et al. 2019). Furthermore, texture is a cross-modal feature that is often linked with haptic perception. Indeed, much of the early work in the perceptual field has examined textured object categorisation on the basis of tactile information. Previous research suggests that tactile exploration of textures, as well as haptic texture matching tasks, are linked with significant activation of the primary (S1) and secondary (S2) somatosensory areas, as well as the parietal operculum, left frontal cortex, medial occipital gyrus (MOG), and bilateral posterior insula (Sathian et al. 2011; Simões-Franklin et al. 2011; Stilla and Sathian 2008; Whitaker et al. 2008b). Importantly, we can distinguish textured information such as smooth or rough using haptic, visual or even auditory cues. Moreover, haptic information can modulate incoming visual information even at very early stages of processing (Eck et al. 2013; Hiramatsu et al. 2011; Lunghi et al. 2010). Likewise, auditory feedback during tactile exploration has been shown to modulate tactile sensation in real time, such that the perceived textures of tactile-explored stimuli (e.g., skin) rely on, specifically, the frequency of the accompanying auditory cues (Guest et al. 2002; Jousmäki and Hari 1998; Yau, Hollins, et al., 2009; Yau et al. 2009). Over time, experience with cross-modal features may allow the visual system to identify textures based on prior knowledge from touch alone (*feeling* velvet predicts visual perception of smoothness), without necessarily requiring corroborating sensory evidence from tactile cues. Despite this, visual and tactile cues for texture perception do not show multisensory benefits, relative to unimodal perception. Instead, each modality seems to encode texture information specific to its unique physiology, and provides the brain with different textural features (Guest and Spence 2003; Heller 1982; Jones and O’Neil 1985; Lederman and Abbott 1981; see Whitaker et al. 2008b; for a review). It is therefore not particularly surprising that we found evidence of activity in CoS – a region which codes for visual texture information – and not regions previously identified to code for haptic texture, during our authenticity categorisation task (Cant et al. 2009; Kravitz et al. 2013; Whitaker et al. 2008b). In order to further investigate the role that textural differences play in driving our ability to distinguish real from fake items, future studies could have participants judge the authenticity of intact and scrambled versions of the images used in this study. If low-level features (e.g., texture) of the objects are necessary for distinguishing real from fake objects, then participants should continue to perform with high accuracy for the scrambled objects. Furthermore, future studies could consider using representational similarity analyses (RSA) to isolate the effects of texture or colour on the categorisation of objects as real or fake..

Previous studies suggest that some regions of the brain appear to respond to both visual and tactile inputs, including the posterior visual cortex and lingual gyrus (Sathian et al. 2011; Stilla and Sathian 2008), as well as through activity in the ventromedial temporal cortex (which are spatially distinct during visual and haptic texture perception, respectively; Podrebarac et al. 2014). While there is evidence to suggest that haptic texture information is associated with activation in early occipital regions or in other regions of the visual cortex (e.g., Amedi et al. 2001; Gallivan et al. 2014; Sathian et al. 2011; Stilla and Sathian 2008), evidence for visual inputs directly affecting activation in somatosensory regions during haptic texture processing is less forthcoming (Eck et al. 2013). For instance, in non-human primates, cells in somatosensory cortex become responsive to visually-presented textures after cross-modal learning (Zhou & Fuster, 1997, 2000). In humans, one study showed that familiar objects categorised visually recruited both primary and sensory cortices (Smith and Goodale 2015). With regards to textures more specifically, another study showed evidence of activation in S2 during visual presentation of different textures – but not when participants were asked to imagine those same textures (Sun et al. 2016). The researchers suggest that this type of activation might support motor planning (e.g., required grip strength for a slippery-looking object). Taken together, these studies provide some evidence that visual texture processing may recruit somatosensory regions as well and may account for some of the activation we observed in the postcentral gyrus (Corkin et al. 1970; Iwamura 1998) and paracentral lobule (Overduin and Servos 2004; Willoughby et al. 2021). Indeed, activation in somatosensory regions of the brain when viewing images of objects could suggest the possibility of cross-modal, visual-tactile activation when evaluating the authenticity of a presented image (e.g., imagining feeling the object’s texture). Given the extensive nature of feedback loops along the ventral visual stream (Smith and Muckli 2010) during tasks including the visual processing of texture information, future studies could consider the timescale of these events - particularly considering that we know little regarding the temporal aspects of visual texture processing (Komatsu and Goda 2018). In particular, elucidating the temporal dynamics could help assess the extent to which activity in early visual cotrices are driving authenticity categorisation performance, or whether later regions involved in object recognition play a larger role.

## Conclusion

In this study, we investigated the neural basis of object categorisation, as it pertains to discriminating real (i.e., natural) from fake (i.e., synthetic) objects. Our results provide evidence suggesting that surface texture is a particularly important determinant to visual object categorisation based on authenticity, as realised in differential activity in the collateral sulcus. Likewise, our results revealed that colour may also be involved in the discrimination of real from fake objects. Colour may have been particularly informative as it has previously been shown to support material identification (Baumgartner et al. 2013; Fleming 2014; Fleming et al. 2013), which in turn may support authenticity discrimination (e.g., brightly or uniformly coloured materials are more likely “fake” than “real”). We also found activity in the lateral occipital cortex, as well as in some somatosensory regions (namely, paracentral lobule and post central gyrus) suggesting the possibility of cross-modal interactions in object identification. Furthermore, our results lend support to previous findings showing correlates in early visual regions – particularly primary visual cortex – when encoding coloured relative to greyscale images of objects.

Our results provide preliminary evidence suggesting that objects categorised as real or fake via only visual input, likely rely on early visual regions of the brain. In particular, activation in regions involved in the processing of texture information, such asthe CoS was associated with object categorisation of based on its authenticity. In turn, the CoS was supported by additional visual regions which may also facilitate texture identification such as those specialised in colour processing. Intruigingly our results also suggest a role for somatosensory regions of the brain although further research is needed to elucidate the specific contribution of this activation to object categorisation.

## Electronic supplementary material

Below is the link to the electronic supplementary material.

Supplementary Material 1

Supplementary Material 2

## Data Availability

Data are available upon request.

## Footnotes

1 Images of flowers, fruit and desserts were combined for analysis, see previous section.
