# Supplementary material for: Visual categorisation of images of familiar objects based on their authenticity: an fMRI study
Source: Exp Brain Res. 2025 Mar 10;243(4):87. doi: 10.1007/s00221-024-06989-3 (PMC11893674; doi:10.1007/s00221-024-06989-3)
Supplement: Supplementary file 1 — Supplementary Material 1 [file 221_2024_6989_MOESM1_ESM.docx]

**Supplemental Materials**

The stimuli used in this study were a series of photographed objects, that were taken from Sharan and colleagues (2014). In a separate pilot study, 16 participants – independent from those in the main study – rated each of these images from one to seven, with a score of one representing ‘very synthetic’ and seven, ‘very natural’. The ratings of this study were subjected to a 2 x 2 x 3 within-subjects ANOVA for ‘authenticity (fake vs. real), colour (colour vs. B&W) and image type (fruit vs. flowers vs. desserts) in SPSS 23.0 (Statistical Package for the Social Sciences by IBM-Corp, Released 2015). The results revealed a significant main effect of authenticity (*F*(1,15)=43.36, *p*<.001, η^2^=.743), where images of fake objects (*M*=3.41, *SD*=0.73) were judged to look more synthetic than real objects (*M*=4.55, *SD*=.41)^^[[1]](#footnote-1)^^. No main effects of colour (*F*<1) or stimulus type (*F*(2,30)=2.73, *p*>.05) were found. There was a significant interaction between authenticity and colour (*F*(1,15)=6.74, *p*<.05, η^2^=.31) but no authenticity by stimulus type interaction (*F*<1), no colour by stimulus type interaction (*F*(2,30)=1.689, *p*>.05) and, finally, no three-way interaction between the factors (*F*(2,30)=2.79, *p*>.05). Post hoc analyses of the interaction between authenticity and colour was unable to determine the source of the interaction as it was discovered that real images were rated higher than fake, regardless of the colour (colour: *F*(1,15)=38.07, *p*<.001, η^2^=.717; B&W: *F*(1,15)=39.49, *p*<.001, η^2^=.725). Therefore, it was deemed that, regardless of colour or image type (i.e., fruit, flowers, or desserts) participants were able to correctly identify images that contained depictions of natural (real) or synthetic (fake) objects. Thus, images of fruit, flowers and desserts were combined for later analysis of the functional neuroimaging data to increase statistical power.

1. Where lower scores reflect a more synthetic, or “fake”, rating. [↑](#footnote-ref-1)
